# Chaperone functional specificity promotes yeast prion diversity

**DOI:** 10.1371/journal.ppat.1006695

**Published:** 2018-01-04

**Authors:** Andrea N. Killian, Justin K. Hines

**Affiliations:** Department of Chemistry, Lafayette College, Easton, Pennsylvania, United States of America; Washington University School of Medicine, UNITED STATES

## Yeast prions and chaperone-dependent propagation

While prions are protein-based infectious agents, yeast prions are protein-based genetic elements of the baker’s yeast *Saccharomyces cerevisiae* [1]. Most yeast prions are amyloid protein aggregates that spread during mitosis through the cytosolic transmission of small, self-templating pieces called propagons. Propagons continue to recruit free protein monomers, perpetuating the prion phenotype in daughter cells [2]. Similar to the reliance of viruses upon host replication machinery, propagation of yeast prions to subsequent cell generations is dependent upon the fragmentation of aggregates by a core set of cellular chaperone proteins to create new propagons. The following three proteins make up the core “prion–chaperone machinery”: the hexameric disaggregase Hsp104, the cytosolic Hsp70 Ssa, and the Hsp40 (also and hereafter called a “J protein”) Sis1 [2]. Propagon generation is dependent upon the severing of amyloid fibers by Hsp104, which requires the upstream action of the Hsp70 Ssa and Sis1 to first bind to amyloids and unfold a portion of the protein, either exposing it or directly transferring it to Hsp104 [3–8]. Here, we will focus on the role of J proteins in promoting the propagation of a wide variety of yeast prions with the aim of better understanding how amyloid diversity is dependent on diverse chaperone activities.

## Yeast prion structures are diverse and chaperone requirements are heterologous

Prions can also form distinct amyloid structures (structural polymorphisms) called “strains” in mammalian systems and “variants” in yeast [2, 9]. These polymorphisms dictate species transmission barriers and disease pathology in mammals, and the intensity of prion-associated phenotypes and stability in mitosis in yeast [9–13]. Recently, we and others have demonstrated that prion–chaperone requirements are heterogeneous and, in contrast to Hsp104 and Hsp70, which have general roles, J proteins appear to represent a prion-specific component of the prion propagation machinery. Most notable has been a direct demonstration that the persistence of distinct prion variants is dependent on the action of different molecular chaperones, of which here we will focus exclusively on the J-protein component. These findings suggest that distinct amyloid structures have unique features that are differentiable by chaperone proteins, revealing a previously unappreciated level of additional complexity that may be exploitable for therapeutic intervention.

## Requirements for Sis1 J-protein activity are distinct and sometimes mutually exclusive

**Fig 1:**
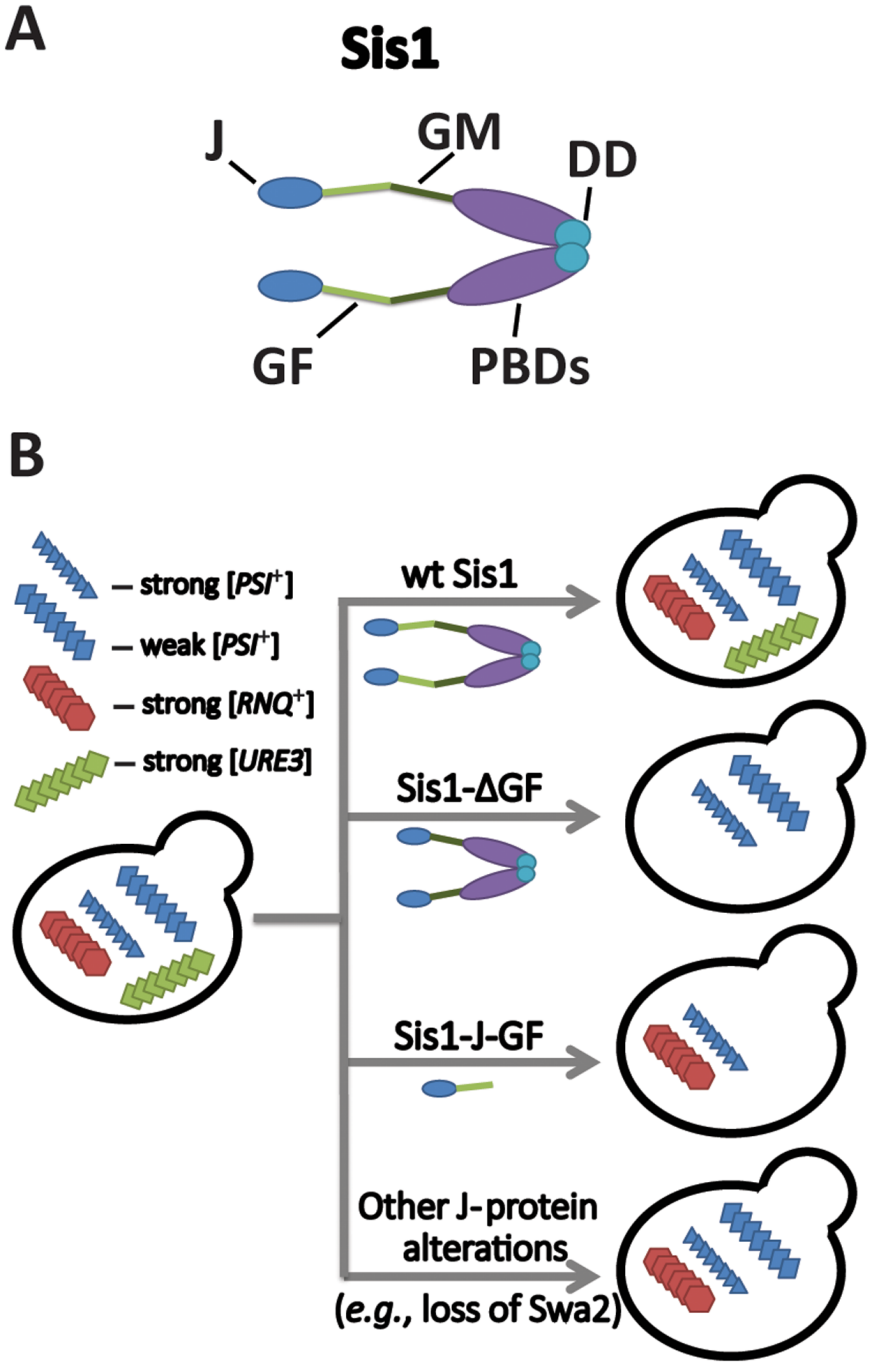
Distinct J-protein functions promote amyloid diversity. (A) Sis1 has five distinct regions denoted using the following notation: DD, dimerization domain; GF, glycine and phenylalanine-rich region; GM, glycine and methionine-rich region; J, J domain; PBDs, peptide-binding domains [14]. (B) Different prions can be selected for or against depending on diverse and sometimes mutually exclusive Sis1 requirements. A construct of Sis1 lacking the GF region (Sis1-ΔGF) maintains all variants of [*PSI*^+^] but not [*RNQ*^+^] or [*URE3*] [7, 21, 22, 26, 27]. Contrastingly, a construct of Sis1 consisting of only the J domain and GF region (Sis1-J-GF) is sufficient to maintain cell viability and to propagate some variants of [*RNQ*^+^] and strong but not weak variants of [*PSI*^+^] [21, 22, 44], demonstrating the existence of mutually exclusive Sis1 requirements with regard to weak [*PSI*^+^] and [*RNQ*^+^]. Finally, either alteration of Sis1 results in the loss of [*URE3*], but deletion of the J protein Swa2 results in loss of [*URE3*] alone [26, 33, 34], demonstrating that novel secondary J-protein requirements exist for some prions. Likewise, but omitted for clarity, the prion [*SWI*^+^] also exhibits a specific requirement for the J protein Ydj1, whereas all three other prions shown in the figure continue to propagate in a strain lacking Ydj1 [7, 24, 29, 33]. This figure is intended to illustrate the possible prions that could propagate in various cells lacking certain chaperone functions. It does not imply that all of these prions and prion variants have been simultaneously observed in a single yeast cell. Although yeast strains can harbor multiple different prions simultaneously, they are often antagonistic toward one another [49], and weak and strong variants of the same prion are not stable due to competition [50].

J proteins act by stimulating Hsp70 ATPase activity, in turn enhancing client peptide binding [14]. Most J proteins can also bind polypeptides directly and deliver them to Hsp70s, allowing J proteins to act as specificity factors, directing and diversifying Hsp70 function [14]. At least four yeast prions rely on the essential J protein Sis1 for stable propagation—[*PSI*^+^], [*URE3*], [*SWI*^+^], and [*RNQ*^+^] (also called [*PIN*^+^]) [15–20]—but the specific requirements for Sis1 activity vary significantly among both prions and prion variants. Mutually exclusive Sis1 requirements have been observed between some prions, specifically between a weak variant of [*PSI*^+^] called [*PSI*^+^]^Sc37^ and a strong variant of [*RNQ*^+^] called [*RNQ*^+^]^STR^; even when experiments are conducted in a single strain initially maintaining both prions, either prion can be selected at the expense of the other depending upon which construct of Sis1 is expressed (see Fig 1 and legend for additional details) [21]. Another recent investigation using multiple variants of [*RNQ*^+^] reached a similar conclusion, finding highly variable, and again sometimes mutually exclusive, Sis1 requirements among variants [22]. Together, these observations suggest that Sis1 has at least two biochemically distinct functions in prion propagation that allow for the propagation of distinct prion variants that are lost from the cell population when the specific Sis1 activity is disrupted. Additionally, despite the fact that prion formation and prion propagation are biochemically distinct processes, a particular variant cannot return to a population if a specific function necessary for its stable propagation is absent. The logical conclusion is that the diversity of chaperone functions grossly limits the number of possible amyloid structures that can be generated and propagated in a given yeast strain.

## Additional J proteins are required by some prions

In addition to Sis1, 12 other J proteins at least partially inhabit the yeast cytosol [23]. While only Sis1 is essential for propagation of strong variants of [*PSI*^+^] and [*RNQ*^+^] [7], an investigation of the prion [*SWI*^+^] revealed that it additionally requires the function of the C-terminal peptide-binding domains of Ydj1, the most abundant J protein in the yeast cytosol [24]. Recent findings have demonstrated the importance of the C-terminal domains of Sis1 and Ydj1 to direct the Hsp70 Ssa to distinct cellular targets and to improve the efficiency of prion fragmentation [21, 25–28]. Although to date there is no direct evidence indicating a specific biochemical role, one hypothesis is that Ydj1 may provide additional access points for Ssa to initiate productive [*SWI*^+^] propagon fragmentation by virtue of direct binding to [*SWI*^+^] aggregates via these domains [24, 29].

A similar investigation of the prion [*URE3*] revealed an essential role for the J protein Swa2, the yeast ortholog of the mammalian protein auxilin, normally responsible for initiating the disassembly of the clathrin lattice on clathrin-coated vesicles following endocytosis [30, 31]. Swa2 is a multidomain protein with clathrin-binding and ubiquitin-associated domains, a tetratricopeptide repeat (TPR) domain, and a J domain [31, 32]. Like auxilin, Swa2 recruits the Hsp70 Ssa to clathrin and stimulates its ATPase activity [30, 31], but interestingly, Swa2’s clathrin-binding domains are not required to propagate [*URE3*], indicating that Swa2’s mechanism of action in prion propagation is independent of its role in mediating clathrin dynamics [33]. Rather, only Swa2’s J domain and TPR domain are required, and although the physiological role of the TPR domain is unknown, TPR domains typically facilitate protein–protein interactions. We recently suggested that this domain may functionally interact with both Hsp70 and Hsp90 in [*URE3*] propagation, forming a novel multichaperone complex to increase prion fragmentation efficiency [34].

## The physical basis for diverse chaperone requirements

The emerging picture is that domains of Sis1 and other J proteins can coordinate in alternate ways to maintain specific prions and prion variants. These divergent J-protein functions are most likely either alternate ways to bind and recruit Hsp70 to aggregates or alternate mechanisms of regulating Hsp70 activity to accomplish prion fragmentation. It seems reasonable to expect that distinct amyloid structures, even formed from the same protein, may interact quite differently with chaperone proteins, requiring a diversity of chaperone functions to accommodate them. For example, work from the True laboratory suggests that [*RNQ*^+^] variants present distinct surfaces for chaperone binding [11, 35].

Notwithstanding the importance of amyloid variant structure, another hypothesis explaining broad differences in chaperone sensitivity between some prions is that the amino acid composition of the prion-forming domain (PrD) of the composite protein is largely responsible. For example, the PrDs of Ure2 and Swi1, the proteins that form [*URE3*] and [*SWI*^+^], are vastly dissimilar from those of Rnq1 and Sup35, which form [*RNQ*^+^] and [*PSI*^+^] [24, 29, 36]. The PrDs of Rnq1 and Sup35 are enriched in glutamine (Q) relative to asparagine (N), rich in glycine and tyrosine, and sparse in certain hydrophobics (F, W, L, I, V, and M) when compared to the PrDs of Ure2 and Swi1 [29]. Others have determined that N-rich sequences are more likely than Q-rich sequences to form amyloids in vivo, though the potential impact of these differences on prion propagon number is unknown [37–40]. Likewise, the presence of tyrosine residues has been suggested to directly promote chaperone-mediated amyloid fragmentation of [*PSI*^+^] and polyQ aggregates [41–43], and the abundance of tyrosines is highly correlated to prion propagon number among strong variants of these four prions [29].

These observations led us to speculate that perhaps both the variations in heritable prion propagons per cell and sensitivities to ectopic chaperone expression found among yeast prions may be primarily dictated by the amino acid composition of the prion protein [29]. For example, N-rich prion aggregates may be more stable in vivo and therefore may require greater intervention by chaperones for fragmentation to keep up with cell division, whereas PrDs devoid of tyrosine might present fewer chaperone-binding sites or potentially decrease the processivity of Hsp104. Indeed, both [*URE3*] and [*SWI*^+^] exhibit low propagon numbers and relatively large intracellular aggregates, indicating that it may be difficult to productively fragment aggregates of these prions to produce propagons relative to other prions. [*URE3*] and [*SWI*^+^] may then require additional chaperone complexes to increase sites of fiber fragmentation for continued propagation, explaining their secondary J-protein requirements, although some sensitivities to subtle changes in chaperone function may simply be a consequence of reduced propagon numbers alone. However, these hypotheses are based on a limited data set, so further examination of additional prion–chaperone interactions is necessary to resolve them.

## J-protein functional diversity scales with organismal complexity

Nearly a dozen amyloid-forming yeast prions have now been identified, but the basic chaperone requirements of most remain unknown, and investigations regarding the role of Hsp70 and its cochaperones in prion propagation have only been conducted for four [3, 28, 29, 36]. Given the significant diversity already apparent, it seems highly likely that other mechanisms of chaperone-dependent, or -independent, amyloid propagation exist and await discovery both in yeast and, notably, in other organisms as well. J proteins like Sis1 and its orthologs have served as a logical entry point for investigating these interactions—Sis1 orthologs from humans (Hdj1/DNAJB1), flies (Droj1), and plants (atDjB1) have been found to support the propagation of some variants of [*PSI*^+^], [*RNQ*^+^], or both when expressed in place of Sis1 in yeast [21, 44]. In general, the number of J-protein genes scales with organismal biochemical complexity; while *S*. *cerevisiae* has 23 and humans have 41, the model plant *Arabidopsis thaliana*—concordant with the large genomes found in plants—has 106, with eight plausible proteins orthologous to Sis1 [45]. One *Arabidopsis* ortholog of Swa2 was even shown to functionally replace Swa2 in the maintenance of [*URE3*] [45]. Considering the significant conservation of function and expanded breadth of J-protein diversity in *A*. *thaliana* and the recent discovery this year that an *A*. *thaliana* protein can propagate as a prion when expressed in yeast [46], the possible discovery of bona fide prions in plants seems ever more likely.

## Conclusions and future directions

Because J proteins often act as targeting factors for Hsp70s, they may constitute the first chaperone response to the presence of amyloid. Due to the general requirement for Sis1 by all studied yeast prions [25], the fact that Sis1 can direct bacterial chaperones to propagate prions in yeast, [24] and its tendency to bind a variety of nonprion amyloids in vivo [47, 48], we speculate that Sis1 may be a general amyloid recognition factor recruiting Hsp70—and vicariously other chaperones like Hsp104—to prion aggregates and other amyloids. Perhaps secondary J-protein requirements exist specifically for amyloid structures that, due to a combination of amino acid composition and three-dimensional structure, are poorly fragmented by the “core” set of chaperones, resulting in larger and less numerous propagons that require additional intervention for efficient fragmentation. A developing theme is that differences in amyloid structure, probably arising from differences in amino acid composition and/or sequence, may create distinct challenges for prion transmission that are overcome by specific J-protein functions that effectively buffer prions against loss during mitosis. Because the elimination of specific chaperone activities leads to selective prion loss, the full complement of J-protein function is required for the persistence of amyloid diversity.
